# Supplementary figures and images for: Long Term Outcome and Quality of Life of Intracranial Meningioma Patients Treated with Pencil Beam Scanning Proton Therapy
Source: Cancers (Basel). 2023 Jun 7;15(12):3099. doi: 10.3390/cancers15123099 (PMC10296362; doi:10.3390/cancers15123099)

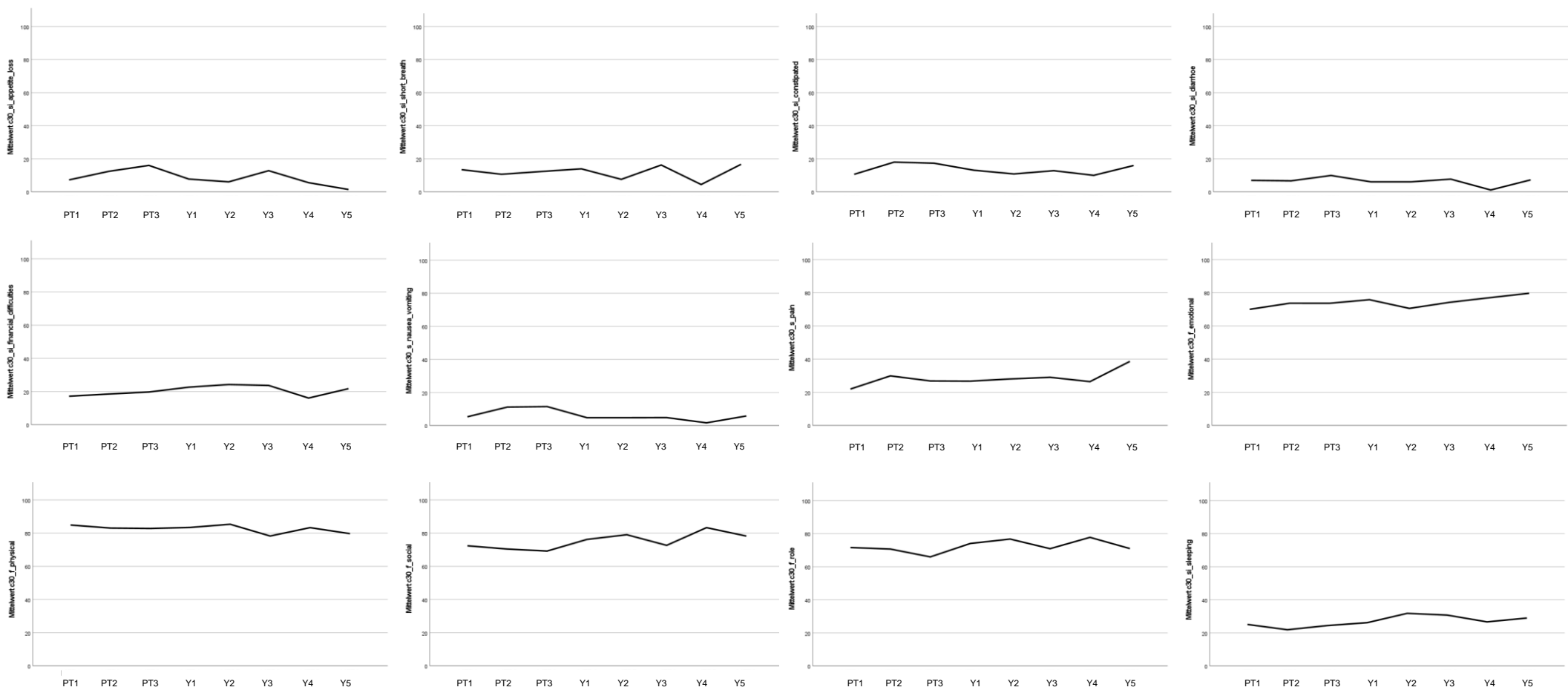

Figure S1: Remaining QLQ-C30 results.

Supplement: Supplementary file 1 [file cancers-15-03099-s001.zip › Figure S1.pdf]

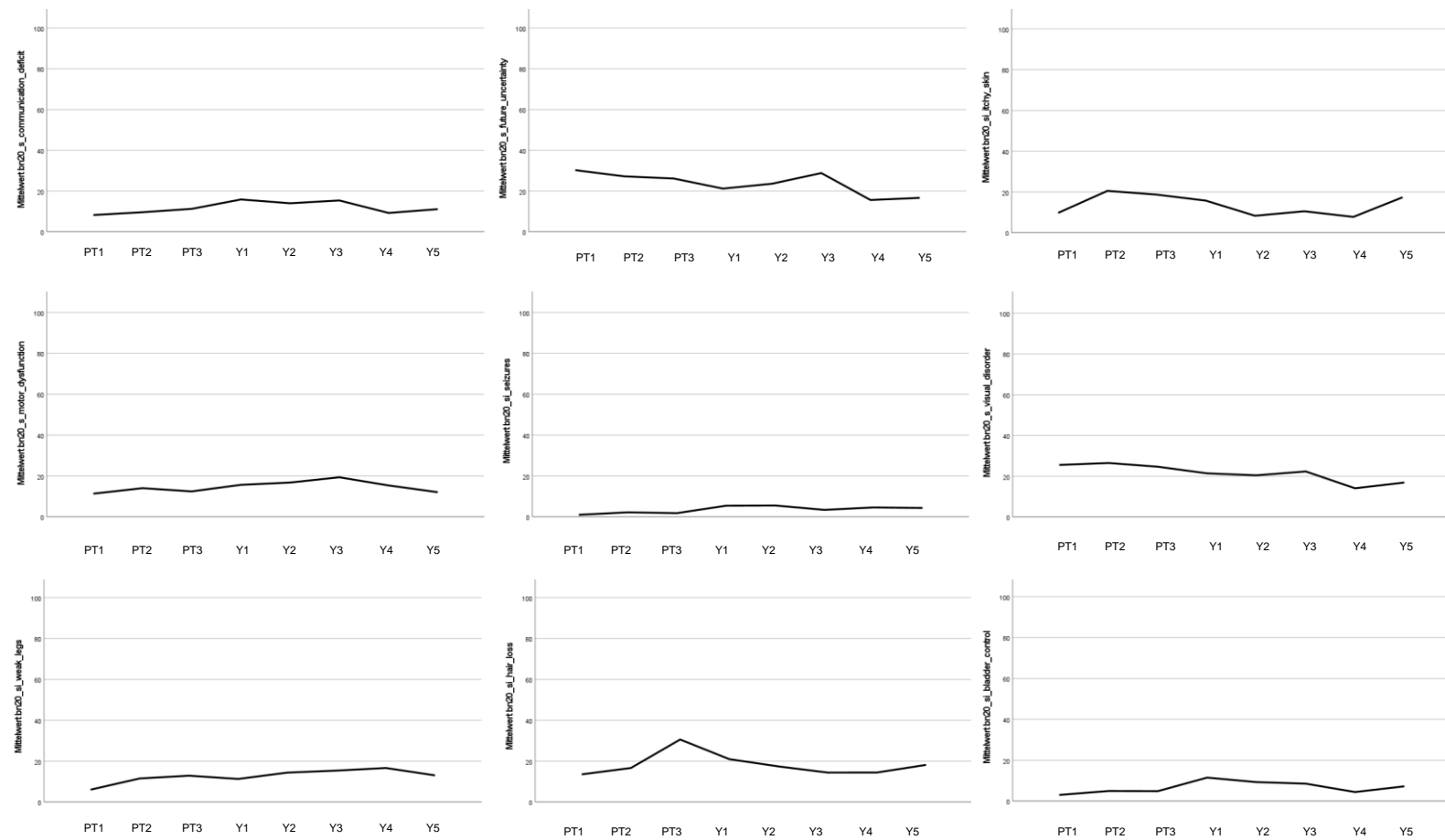

Figure S2: Remaining QLQ-BN20 results.

Supplement: Supplementary file 1 [file cancers-15-03099-s001.zip › Figure S2.pdf]
